# A Survey on the Knowledge and Awareness of Testicular Cancer and Testicular Self-Examination Among Men in Saudi Arabia

**DOI:** 10.7759/cureus.55778

**Published:** 2024-03-08

**Authors:** Khalid Altowaijri, Mohammed Aldehaim, Othman Alshammari, Rakan Aldohan, Fayez AlTabbaa, Ambreen Kazi

**Affiliations:** 1 College of Medicine, King Saud University, Riyadh, SAU; 2 Family and Community Medicine, King Saud University Medical City, Riyadh, SAU

**Keywords:** saudi arabia, sources of information, education, testicular self-examination, testicular cancer

## Abstract

Objectives

The aim of the study was to assess the knowledge and awareness of testicular cancer (TC) and testicular self-examination (TSE) and to identify the associated factors in men in Saudi Arabia.

Methods

An online questionnaire-based study was conducted in Saudi Arabia with a representative sample of 794 participants. The questionnaire comprised knowledge, awareness and attitude questions regarding TC and TSE in addition to signs/symptoms and risk factors. Multivariate logistic regression analysis was conducted to identify the significant variables associated with knowledge of TC and TSE.

Results

Around 43% (n=340) of the participants had inadequate knowledge of TC, whereas 26% (n=205) had heard about TSE and only 65 (8.2%) performed TSE. The first model for knowledge found that participants with a low level of education [2.75 (1.18, 6.42)]; no past history of a testicular problem [2.20 (1.22, 3.95)] and those who had not heard about TSE [1.79 (1.24, 2.57)] were at higher odds for inadequate knowledge, whereas those whose mothers had received college-level education [0.39 (0.19, 0.79)] and those who received information from school/college [0.61 (0.37, 0.97)] were more likely to have adequate knowledge about TC. The second model for TSE found that a low level of education 5.24 (1.34, 20.52) was associated with not performing TSE. Receiving information from social media [0.08 (0.03, 0.17)], school/college [0.06 (0.02, 0.13)], family and friends [0.17 (0.05, 0.57)] and medical staff [0.08 (0.03, 0.17)] were associated with higher odds of performing TSE.

Conclusion

The majority of Saudi males have knowledge about TC. On the contrary, only a small percentage of the respondents have heard of or performed TSE as a screening technique. Educated sources of information can be a reliable way of giving correct knowledge on sensitive topics like TSE.

## Introduction

Testicular cancer (TC) is a malignant tumour of the testicle, which is responsible for the secretion of testosterone. It is considered the most common malignancy in young males with an age range of 15-45 years [1]. The survival rate is higher for people diagnosed with early-stage cancer and lower for those with late-stage cancer. For TC that has not spread beyond the testicles (stage 1), the survival rate is more than 96% five-year survival [1]. The survival rate for TC is 73% when it spreads outside the testicles to regions past the lymph nodes, like the lungs or other organs [2].

A study revealed a correlation between the stage of the TC tumour and patient delay diagnosis [3]. Early detection is the most effective strategy to reduce TC mortality [4]. There is also evidence that TC diagnosis and treatment may cause psychological problems in patients such as anxiety, distress due to infertility, and fear of recurrence, all of which decrease overall life satisfaction and also may affect social contacts and family relationships [5-7]. Testicular self-examination (TSE) is an easy screening technique that involves inspection and palpation of the testes to detect any changes for early detection of TC. In this procedure, males check their own testicles in order to rule out any unusual lumps or bumps, which may be the first sign of TC [8].

A total of 8430 new cases of TC were reported globally in 2015 [9]. Based on the Saudi cancer registry, the national prevalence of TC accumulated 1526 cases over a 20-year period (1994-2013). In the first decade (1994-2004), the incidence rate of TC was approximately 30.5 cases annually; however, the second decade (2004-2013) had a consistently considerable increase in the incidence rate, with a mean of 70 cases annually [10].

According to TSE, a study was conducted, in 2021, in Turkey with 345 participants, 174 of them were medical students and 171 were control group. The study found that awareness in medical students about TSE was 52.3% and 23% in the control group [11]. Another study conducted in Poland in 2022 with 771 participants found that only 52.4% of the participants had performed TSE [12].

Although incidence rates varied greatly by geographical region, Western Europe, Northern Europe, and Australia/New Zealand had the highest incidence [13]. Although a high incidence was noted in Western and Northern Europe, the mortality rate was relatively low, suggesting the beneficial effects of a prompt diagnosis followed by effective multimodal treatment and surveillance [13]. However, whereas the overall incidence of TC was low in Africa and Asia, mortality rates roughly equalled incidence rates, likely because of the lack of effective tools for diagnosis and treatment [13].

In addition, limited knowledge contributes to late diagnosis, detection and reporting of cases. A cross-sectional study from Bahrain comprising 243 males reported that approximately 47% of the participants were not aware that men can get TC. More than 80% perceived that they had low-risk factors for getting TC, and only 1% answered that their risk for developing TC was high. Only 12% answered correctly to the common symptoms of TC. Nearly 80% of the participants had not heard about TSE, and less than 10% knew how to perform it. Only 5.8% reported that they previously performed TSE [14]. A study was conducted in Turkey comprising 275 male university students aged from 20 to 25 years. The study found that 88% of its participants did not have previous knowledge about TSE [15]. Another study from Nigeria concluded that 88.6% of its respondents had not heard about TC and only 1% of the participants were aware or had previous knowledge about TSE [16]. A study conducted in Poland concluded that 71.9% of high school students and almost 100% of medical students correctly identified the term “testicular cancer”, however, for epidemiological facts both study groups scored very low, but a greater number of correct answers came from medical students. The answers regarding TSE scored very low. In addition, over 80% of high school students and over 50% of medical students admitted they never performed TSE. Only 9.9% of high school students and 30% of medical students practised TSE at least once a month [17].

All the data above show the importance of correct knowledge about TC and TSE. The incidence rate in the last decade has more than doubled in Saudi Arabia [10]. So hopefully this study will help in spreading awareness of TC and TSE and diagnose TC in early stages. Early detection of any cancer is important because it will be easier to treat, prevent it from spreading, and have a better prognosis [18]. Men's total testicular health, self-awareness, and well-being can be ensured by promoting TSE practices [19]. There are however several risk factors that increase the likelihood of developing TC, and these risk factors can be divided into unmodifiable (such as HPV infection, family history of TC and cryptorchidism) and modifiable risk factors (such as sociodemographic factors related to age, occupation, BMI, lifestyle and testicular heat exposure) [20-23].

There is indeed insufficient data in Saudi Arabia that assesses knowledge levels of TC and awareness of TSE habits among Saudi men; this fact emphasizes why we need to conduct this study. Our study had two objectives, the first was to determine the levels of knowledge and awareness about TC among men who live in Saudi Arabia. The second objective was to identify the significant socio-demographic factors and sources of information associated with the knowledge about TC and TSE in men in Saudi Arabia.

## Materials and methods

A cross-sectional study was conducted in Saudi Arabia using an online survey format through the Google Forms platform. In addition, forms were distributed online using personal contacts. The data were collected from August 2021 to November 2021. The inclusion criteria were all Arabic-speaking men, aged 18 years and above, living in Saudi Arabia. All men who were suffering from scrotal or testicular problems were excluded from the study. The sample size was calculated based on the assumption that 50% of the participants have correct knowledge about TC and keeping the alpha level of 0.05, and confidence interval (CI) at 95%; 450 male participants were required to estimate the population having correct knowledge. To measure the association between sociodemographic factors and knowledge, assuming a type-I error of 0.05, type-II error of 0.20 (power of 0.80) and a 30% difference between the two groups, we needed 832 participants. Assuming, after discarding the incomplete forms, 794 participants were included in the final analysis.

Data collection was based on a questionnaire composed of four sections. The first section included sociodemographic information including age, cities and marital status, and the participants' and parents' levels of education and occupation. The second section included questions about sources of information and signs and symptoms for TC (school or college, friends or family, social media, medical staff), as well as signs and symptoms (painless lump, sensation of heaviness in the scrotum, and dull ache in the lower abdomen) and question about the definition TC. The third section comprises lifestyle variables including smoking, alcohol consumption, and sexual activity, as well as risk factors like age, family history, undescended testicles and mumps. The fourth section consisted of questions on knowledge and practice about TSE and the sources of information. The questionnaire was developed in English, then translated into Arabic and back-translated. It was reviewed by senior researchers and family physicians to clear any ambiguity or duplication. Pretesting was conducted on a separate sample of 30 individuals. According to the literature, the correct signs and symptoms of TC are a painless lump, sensation of heaviness and dull ache [1,24]. And the risk factors are age group between 15 and 45, family history, undescended testis, presence of mumps, smoking and alcohol consumption and fertility problems [15,22,25-27].

Data analysis

The data were analyzed using the Statistical Package for the Social Sciences (IBM SPSS Statistics for Windows, IBM Corp., Version 22.0, Armonk, NY). The variable “Knowledge about Testicular Cancer” (TC) was based on the question related to what is TC and the correct signs and symptoms. A composite variable was developed based on seven questions, which were further categorized based on the mean cut-off value. Participants scoring ≥5 were labelled as those with adequate knowledge and coded as “0”, versus those who scored <5 were labelled as “inadequate knowledge” and coded as 1. Descriptive analysis was conducted and frequency with percentages were calculated for the categorical variables. The Chi-square test and p-value were used to measure the association between sociodemographic variables, personal history, and risk factors with the knowledge of TC and TSE. The p-value was kept significant at <0.05. Multivariate logistic regression was conducted to identify the significant factors associated with knowledge about TC and TSE by calculating the adjusted odds ratio and 95% CI. Two independent models were developed, first for the factors associated with inadequate knowledge of TC and second model to identify the significant factors associated with lack of TSE.

Ethical considerations

All the information that was collected was for research purposes only. The participant’s anonymity was maintained by giving a unified identification number to each study participant. The questions were phrased according to the cultural values and norms. The study proposal was reviewed and approved by the ethical committee under the Institutional Review Board (IRB), King Saud University, Riyadh, Saudi Arabia.

## Results

In total, 794 people participated in the online survey from different regions of Saudi Arabia. The sociodemographic profile of the participants showed that the majority were single males, between the ages of 18 to 24 years, college/university-going students, and having an educated and employed father. Around 20% of the participants were cigarette smokers.

The mean score for the knowledge variable was 5.0 (±1.10) and ranged from 0 to 7. Only 2.5% (n=20) of participants correctly answered all the questions and scored 7. Based on the median cut-off value of 5.0 for the knowledge variable, more than 57.2% (n=454%) of the participants scored ≥5 (adequate knowledge) whereas, around 42.8% (n=340) scored <5.0 and were labelled as having inadequate knowledge for TC.

Table 1 shows the descriptive statistics for the knowledge variable. TC was correctly defined by 84% of the participants, however, some of the common signs/symptoms, such as pain while urinating (60%), painless swelling (lump) in the testicles (41%), sensation of heaviness in the scrotum (40%), dull ache in the lower abdomen (72%) were incorrectly answered by a significant number of the participants. Similarly, a significant percentage of participants gave incorrect answers for different risk factors for TC, such as the correct age group at risk for TC (45% answered incorrectly), family history of TC (31%), fertility problems and sperm quality (45%), undescended testis (57%), presence of mumps (84%) and ethnicity (98%).

**Table 1:** Frequency and percentage of participants mentioning correct and incorrect signs and symptoms and risk factors for testicular cancer in Saudi Arabia (n=794)

Signs and symptoms and risk factors for testicular cancer in Saudi Arabia	Variable	Frequency n (%)
Heard about testicular cancer	Yes	462 (58.2)
	No	332 (41.8)
Define testicular cancer	Correctly	665 (83.8)
	Incorrectly	129 (16.2)
Knowledge about signs and symptoms		
Pain while urinating	Correct	320 (40.3)
	Incorrect	474 (59.7)
Stomach cramps or bloating	Correct	703 (88.5)
	Incorrect	91 (11.5)
Painless swelling lump in the testicles	Correct	465 (58.6)
	Incorrect	329 (41.4)
Sensation of heaviness in the scrotum	Correct	478 (60.2)
	Incorrect	316 (39.8)
Joint pain	Correct	735 (92.6)
	Incorrect	59 (7.4)
Dull ache in lower abdomen	Correct	226 (28.5)
	Incorrect	568 (71.5)
Total score* Mean (SD) (5.0 (±1.10))	50^th^ percentile	5.0
	75^th^ percentile	5.0
Categorical	Adequate knowledge	454 (57.2)
	Inadequate knowledge	340 (42.8)
*Knowledge was categorized into “adequate” and “inadequate” based on the median cut-off value of 5 determined after calculating the sum for the correct answers related to the definition and signs and symptoms for TC		
Knowledge about risk factors		
18-45 years age group at highest risk	Correct	434 (54.7)
	Incorrect	360 (45.3)
Family history of testicular cancer	Correct	548 (69)
	Incorrect	246 (31)
Fertility problems and sperm quality	Correct	438 (55.2)
	Incorrect	355 (44.8)
Undescended testes	Correct	343 (43.3)
	Incorrect	451 (56.7)
Diabetes	Correct	633 (79.8)
	Incorrect	161 (20.2)
Presence of mumps infection	Correct	124 (15.6)
	Incorrect	670 (84.4)
Hypertension	Correct	695 (87.6)
	Incorrect	99 (12.4)
Low birth weight	Correct	768 (96.8)
	Incorrect	26 (3.2)
Height	Correct	771 (97.2)
	Incorrect	22 (2.8)
Ethnicity	Correct	18 (2.3)
	Incorrect	776 (97.7)

Table 2 shows the univariate analysis for sociodemographic characteristics, medical history and sources of information associated with knowledge variables. Participants with school-level education [2.62 (1.24, 5.54)] and college level [2.69 (1.30, 5.58)] were at higher odds for inadequate knowledge of TC with respect to those with university-level education. Participants who had not heard about TC [1.62 (1.22, 2.16)] and those who had no history of any testicular problems [2.30 (1.30, 4.01)] were at higher odds for inadequate knowledge. A significant percentage of participants (41%) reported that their mothers have college-level education, and they were found to have lower odds [0.44 (0.23, 0.86)] for inadequate knowledge. The most common source of information reported was social media (31%), followed by school/college (13%) and then family/friends (9%). Only 6% mentioned that they received information from medical staff. Except for the family/friend category, participants receiving information from social media, school/college, and medical staff were 0.67 (0.48, 0.94), 0.54 (0.34, 0.86) and 0.43 (0.22, 0.83) at lower odds for inadequate knowledge, respectively. Other variables such as age, father’s education, family history, and smoking were not associated significantly with the knowledge variable.

**Table 2:** Univariate analysis showing factors associated with inadequate knowledge of testicular cancer in men in Saudi Arabia

Variables	Categories	Total N=794	Inadequate knowledge* N = 340 (42.8%)	Adequate knowledge N =454 (57.2%)	Unadjusted odds ratio with 95% CI
Age (in years)	18-24	653 (82)	284 (83.5)	369 (81.3)	1.0
	>24	141 (18)	56 (16.5)	85 (18.7)	0.86 (0.59, 1.24)
Participant’s education	Postgraduate	44 (5.5)	10 (2.9)	34 (7.5)	1.0
	College	502 (63.2)	222 (65.3)	280 (61.7)	2.69 (1.30, 5.58)
	School	248 (31)	108 (31.8)	140 (30.8)	2.62 (1.24, 5.54)
Father’s education	Postgraduate	168 (2%)	67 (19.7)	101 (22.2)	1.0
	College	288 (36)	102 (30)	186 (41)	0.92 (0.62, 1.37)
	School	338 (42)	171 (50.3)	167 (36.8)	1.39 (0.96, 2.01)
Mother’s education	Postgraduate	39 (4.9)	22 (6.5)	17 (3.7)	1.0
	College	325 (40)	118 (34.7)	207 (45.6)	0.44 (0.23, 0.86)
	School	430 (54)	200 (58.8)	230 (50.7)	0.67 (0.35, 1.30)
Participants occupation	Student	522 (65)	227 (66.8)	295 (65)	1.0
	Working	180 (22)	66 (19.4)	114 (25.1)	0.75 (0.53, 1.07)
	Not working	92 (11)	47 (13.8)	45 (9.9)	1.36 (0.87, 2.112)
Father’s occupation	Working	593 (74.6)	248 (72.9)	345 (76)	1.0
	Not working	201 (25.3)	92 (27.1)	109 (24)	1.17 (0.85, 1.62)
Mother’s occupation	Working	264 (33.2)	111 (32.6)	153 (33.7)	1.0
	Not working	530 (66.7)	229 (67.4)	301 (66.3)	1.05 (0.77, 1.41)
Marital status	Single	703 (88.50)	304 (89.4)	399 (87.9)	1.0
	Married	91 (11.4)	36 (10.6)	55 (12.1)	0.86 (.55, 1.34)
Heard about testicular cancer	Yes	462 (58.1)	175 (51.5)	287 (63.2)	1.0
	No	332 (41.8)	165 (48.5)	167 (36.8)	1.62 (1.22, 2.16)
Smoking	Yes	174 (21.9)	66 (19.4)	108 (23.8)	1.0
	No	620 (78)	274 (80.6)	346 (76.2)	1.29 (0.92, 1.83)
Alcohol intake	Yes	36 (4.5)	18 (5.3)	18 (4)	1.0
	No	758 (95.4)	322 (94.7)	436 (96)	1.35 (0.69, 2.64)
History of testicular problem	Yes	66 (8.3)	17 (5)	49 (10.8)	1.0
	No	728 (91.6)	323 (95)	405 (89.2)	2.30 (1.30, 4.01)
Family history of testicular cancer	Yes	19 (2.3)	10 (2.9)	9 (2)	1.0
	No	774 (97.4)	329 (97.1)	445 (98)	0.66 (.27, 1.66)
Source of information	Haven’t heard about it	330 (41)	164 (48.2)	166 (36.6)	1.0
	School/college	103 (12)	36 (10.6)	67 (14.8)	0.54 (0.34, 0.86)
	Family/friends	69 (8)	28 (8.2)	41 (9)	0.69 (0.41, 1.17)
	Social media	245 (30)	98 (28.8)	147 (32.4)	0.67 (0.48, 0.94)
	Medical staff	47 (5)	14 (4.2)	33 (7.3)	0.43 (0.22, 0.83)

Around 26% (n=205) of participants had heard about TSE and only 65 (8.2%) performed TSE. Table 3 is showing the univariate analysis for sociodemographic characteristics, medical history and sources of information associated with TSE. Participants with school-level education [4.0 (1.69, 9.45)] and those with college-level education [3.59 (1.65, 7.83)] were at higher odds of not performing TSE. Participants who had fathers with low school-level education and those who were not unemployed were 1.79 (1.0, 3.33) and 2.23 (1.08, 4.58) times at higher odds for not performing TSE, respectively. Similar to knowledge about TC, participants who had not heard about TC [3.46 (1.82, 6.58)], those not have past history of testicular problems [2.49 (1.23, 5.05)] and those having a negative history for TC [3.12 (1.01, 9.68)] were at higher odds for not performing TSE. Participants reported social media (12%) as the most common source of information for TSE, followed by school/college (6%), family and friends (4.5%) and medical staff (3.4%) as the last. All types of sources were associated with giving information about TSE. Source of information such as social media [0.08 (0.04, 0.16)], school/college [0.05 (0.02, 0.11)], family/friends [0.17 (0.05, 0.55)] and medical staff [0.02 (0.009, 0.06)] were associated with lower odds for not performing TSE.

**Table 3:** Univariate analysis showing an association for socio-demographic characteristics, personal habits, and sources of information with performing testicular self-examination in men in Saudi Arabia

Questions in sociodemographic characteristics, personal habits, and sources of information	Variables	Don’t perform TSE n=729 (91.8%)	Perform TSE n=65 (8.2%)	Unadjusted odds ratio with 95% CI
Age (in years)	18-24	603 (82.7)	50 (76.9)	1.0
	>24	126 (17.3)	15 (23.1)	0.69 (0.38, 1.28)
Participant’s education	Postgraduate	34 (4.7)	10 (15.4)	1.0
	College	464 (63.6)	38 (58.5)	3.59 (1.65, 7.83)
	School	231 (31.7)	17 (26.2)	4.0 (1.69, 9.45)
Father’s education	Postgraduate	148 (20.3)	20 (30.8)	1.0
	College	249 (34.2)	20 (30.8)	1.68 (0.88, 3.23)
	School	332 (45.5)	25 (38.5)	1.79 (1.0, 3.33)
Mothers education	Postgraduate	34 (4.7)	5 (7.7)	1.0
	College	298 (40.9)	27 (41.5)	1.62 (0.58, 4.49)
	School	397 (54.5)	33 (7.7)	1.76 (0.65, 4.83)
Fathers occupation	Working	537 (73.7)	56 (86.2)	1.0
	Not working	192 (26.3)	9 (13.8)	2.23 (1.08. 4.58)
Marital status	Single	647 (88.8)	56 (86.2)	1.0
	Married	82 (11.2)	9 (13.8)	0.79 (0.38, 1.65)
Heard about testicular self-examination	Yes	409 (56.1)	53 (81.5)	1.0
	No	320 (43.9)	12 (18.5)	3.46 (1.82, 6.58)
Smoking	Yes	161 (22.1)	13 (20)	1.0
	No	568 (77.9)	52 (80)	0.88 (0.47, 1.66)
Alcohol intake	Yes	698 (95.7)	60 (92.3)	1.0
	No	31 (4.3)	5 (7.7)	0.53 (0.20, 1.42)
History of testicular problem	Yes	55 (7.5)	11 (16.9)	1.0
	No	674 (92.5)	54 (83.1)	2.49 (1.23, 5.05)
Family history of testicular cancer	Yes	15 (2.1)	4 (6.2)	1.0
	No	713 (97.9)	61 (93.8)	3.12 (1.01, 9.68)
Source of information for testicular self-examination	Haven’t heard about it	573 (78.6)	12 (18.5)	1.0
	Medical staff	14 (1.9)	13 (20)	0.02 (0.009, 0.06)
	Social media	76 (10.4)	21 (32.3)	0.08 (0.04, 0.16)
	Family/friends	32 (4.4)	4 (6.2)	0.17 (0.05, 0.55)
	School/college	34 (4.7)	15 (23)	0.05 (0.02, 0.11)

Table 4 shows the multivariate logistic regression analysis with the adjusted odds ratio and 95% CI for the various factors associated with the participant's knowledge about TC. After adjusting for age, father’s education and occupation and family history the following variables were significantly associated with inadequate knowledge. Participants with college-level education [2.77 (1.23, 6.24)] and school-level education [2.75 (1.18, 6.42)] were at higher odds of having low knowledge. Participants whose mothers had received college-level education were 61 times [0.39 (0.19, 0.79)] more likely to have adequate knowledge of TC. Participants with no past history of testicular problems [2.20 (1.22, 3.95)] and those who had not heard about TSE [1.79 (1.24, 2.57) were at higher odds for inadequate knowledge. Sources of information found that only those who received information from school/college [0.61 (0.37, 0.97)] were 39 times more likely to have adequate knowledge about TC.

**Table 4:** Multivariate logistic regression analysis showing the association between sociodemographic factors and sources of information with poor knowledge of testicular cancer in Saudi Arabia

Questions in sociodemographic factors and sources of information	Variables	Adjusted odds ratio (95%CI)
Age (in years)	18-24	1.0
	>24	0.96 (0.62, 1.51)
Participant’s education	Postgraduate	1.0
	College	2.77 (1.23, 6.24)
	School	2.75 (1.18, 6.42)
Mother’s education	Postgraduate	1.0
	College	0.39 (0.19, 0.79)
	School	0.59 (0.29, 119)
Father’s occupation	Working	1.0
	Not working	1.12 (0.79, 1.57)
Marital status	Single	1.0
	Married	0.97 (0.49, 1.91)
Past history of testicular problem	Yes	1.0
	No	2.20 (1.22, 3.95)
Family history of testicular cancer	Yes	1.0
	No	0.53 (0.19, 1.43)
Heard about testicular self-examination	Yes	1.0
	No	1.79 (1.24, 2.57)
Source of information for knowledge on testicular cancer	Haven’t heard about it	1.0
	Medical staff	0.87 (0.39. 1.92)
	School/college	0.61 (0.37, 0.97)
	Family/friends	0.73 (0.41, 1.31)
	Social media	0.74 (0.52, 1.03)

## Discussion

According to the survey's findings, a significant amount of responders had never heard of TC were unsure of the term's precise definition, and had never heard of TSE. This ignorance is alarming since prompt diagnosis and treatment of TC depend on early recognition and understanding of the condition [18].

The results show that 43% of participants had knowledge scores below 5.0, indicating insufficient familiarity with TC. Furthermore, despite the fact that 84% of respondents could accurately define TC, there were misunderstandings regarding typical indications and symptoms, including lower abdominal dull discomfort, testicular swelling that is painless, heaviness in the scrotum, and pain while urination. Furthermore, a significant number of individuals gave incorrect replies when asked about several TC risk factors, such as ethnicity, mumps, age group at risk, family history, infertility issues, and undescended testis. This implies a lack of knowledge of the variables that lead to TC. This study will help in spreading awareness of TC and TSE and diagnose TC in the early stages. Moreover, a considerable proportion of the participants (about 74%) were unaware of the existence of TSE. Given that TSE is an essential tool for the early identification of TC [17]. This lack of awareness is troubling. Our study is in contrast to a study conducted in 2019, in Southwest of Uganda, that found around 40% of the participants were aware of TSE [8].

The fact that so many participants had never heard of TSE points to a serious lack of information and instruction on this essential self-evaluation. A number of things could be to blame for this lack of knowledge, such as restricted information sharing, the absence of focus on self-examination in public health initiatives, and inadequate education regarding TC in general [17]. All things considered, these results emphasize the necessity of focused educational efforts and awareness initiatives to raise public understanding of TC, including its description, symptoms, risk factors, and the significance of routine self-examinations. We can enable people to take proactive measures toward early detection and improve results against TC by filling in these knowledge gaps [28].

Positive past medical and family history describes situations in which a person or members of their family have dealt with specific illnesses or ailments. This data is useful since it makes genetic predispositions and possible risk factors easier to identify. For instance, a positive family history of heart disease could point to a higher risk for the affected person. It enables medical practitioners to carry out screenings that are suitable, use preventive measures, and modify treatment programs as necessary [23]. Conversely, a negative medical history suggests that neither the individual nor their family members have any significant illnesses or medical conditions. Absolute immunity is not guaranteed, even though it can be comforting to not have a history of certain health problems. To maintain general well-being, preventive measures, healthy lifestyle choices, and routine check-ups are still essential [23].

In general, a positive medical and family history offers vital information for risk assessment and customized care, whereas a negative history implies a lower risk but does not mean preventive care is not necessary. Because it affects the information's credibility and reach, the information source plays a critical role in raising general awareness about TC and TSE.

The most popular method for raising general awareness is at schools. Medical professionals can ensure that young people receive accurate information at an early age and reach a large number of individuals by incorporating TC and TSE education into the curriculum. Important health-related messages can be imparted in an orderly and structured setting in schools, which can have a long-lasting effect on students' behaviour and knowledge [8,17,29]. Social media sites are now effective tools for disseminating information to a wide audience. Medical professionals can use social media to dispel myths about TC and TSE, share success stories, and produce educational content. Social media's interactive features encourage participation and dialogue, which raises awareness and knowledge among the community at large [30,31]. When it comes to educating the youth about TC and TSE, medical personnel are essential. They can dispel misconceptions, increase awareness, and offer correct information through educational campaigns, workshops, and social media outreach. By interacting with patients and their social networks and working with educational institutions, healthcare providers can enable youth to take charge of their own health. By improving knowledge about TC and promoting early detection, medical staff contribute to the overall well-being of the community [16].

Strengths and limitations

The major strength of this study was that the sample was collected from all major regions of Saudi Arabia, thus making our results generalizable. Unfortunately, on account of the pandemic caused by the coronavirus disease (COVID-19), we were not able to conduct a face-to-face survey. Hence, one of the challenges that we faced during the conduction of the study was encountering uncooperative participants, and the limitations associated with the online questionnaire distribution method.

## Conclusions

According to the findings, our study has proved that the majority of Saudi males have knowledge about TC. Whereas, only a small percentage of the respondents have heard of or performed TSE as a screening test. The results indicate that parents should help their children to get educated by a trustworthy source about TC and its symptoms as well as TSE performance.

The health authorities should raise public awareness about the matter in a more intriguing way to the public, such as by conducting public campaigns that encourage medical screening and reporting. We also advise future potential investigators pursuing the same objectives to further explore the factors associated with TC.
